# Screening of Insecticidal and Antifungal Activities of the Culturable Fungi Isolated from the Intertidal Zones of Qingdao, China

**DOI:** 10.3390/jof8121240

**Published:** 2022-11-24

**Authors:** Xiufang Wang, Guixia Ji, Jingfang Cun, Pengjun Xu, Xinwei Wang, Guangwei Ren, Wei Li

**Affiliations:** 1 Key Laboratory of Tobacco Pest Monitoring & Integrated Management in Tobacco, Tobacco Research Institute of Chinese Academy of Agricultural Sciences, Qingdao 266101, China; 2 College of Science, Shantou University, Shantou 515063, China; 3 College of Marine Life Sciences, Ocean University of China, Qingdao 266005, China

**Keywords:** marine-derived fungi, antifungal activity, insecticidal effect, bioactivity, agricultural application

## Abstract

Numerous studies focused on drug discovery perspective have proved the great potential for exploration of marine-derived fungi to seek bioactive chemicals. Yet, marine-derived fungi are less explored compared to their terrestrial counterparts. Here, 181 fungal strains (134 species) isolated from marine algae and sediment in Chinese intertidal zones were screened to reveal bioactivities using brine shrimp, green peach aphid and plant pathogens as targets. Fermentation supernatants of 85 fungal strains exhibited a high lethality (>70%) of brine shrimp at 24 h, and 14 strains appeared to be acute-toxic as featured by more than 75% mortality at 4 h, indicating efficient insecticidal bioactivity. The crude extracts of 34 strains displayed high toxicity to green peach aphid with more than 70% of mortality at 48 h. For the plant pathogens tested, the inhibitory rates of eight fungal strains affiliated with *Alternaria* (AS3, AS4), *Amphichorda* (AS7), *Aspergillus* (AS14), *Chaetomium* (AS21), *Penicillium* (AS46), *Purpureocillium* (AS55) and *Trichoderma* (AS67) were equal or higher than that of the positive Prochloraz, and five of them (AS7, AS14, AS21, AS55, AS67) were also strongly toxic to brine shrimp or aphid. Our findings indicate broad potential for exploration of marine-derived fungi as candidate resources to pursue bioactive compounds in controlling agricultural pests and pathogens.

## 1. Introduction

Compared to terrestrial or freshwater habitats, marine habitats are typically represented by extreme living conditions, including pressure, lower temperature, higher levels of pH, salinity, metals (e.g., Fe, Cu, Mo, Zn, Cd, Pb), etc. [1,2,3]. Fungi in marine environments have developed adaptations to cope with these environmental stresses through expression of specific biosynthetic pathways that lead to diverse secondary metabolites such as polyketides, alkaloids or peptides [4,5]. Many secondary metabolites are used as valuable compounds for therapeutic targets, thus attracting biotechnologists to explore marine biodiversity for novel metabolites and drugs [6]. Cephalosporin C, the first natural product from the marine fungus *Acremonium chrysogenum* and isolated from seawater close to a sewage outlet off the Sardinian coast, was discovered as early as 1946 [7]. So far, more than 3500 secondary metabolites have been isolated from fungi collected from marine environments. According to the *Natural Product Atlas*, secondary metabolites from marine-derived fungi now represent approximately 22% of all metabolites isolated from fungi [8].

Nowadays, issues related to resistance of agricultural pests and diseases and environmental pollution of chemicals have aroused great concern worldwide [9]. Therefore, it is necessary to pursue novel chemicals to solve these issues of pest and disease control. Many studies documented that secondary metabolites from marine-derived fungi have antiphytogenic and insecticidal activities (e.g., [4,6,10]). For example, a novel peniciaculins A was isolated from the deep-sea sediment-derived fungus *Penicillium aculeatum* (strain SD-321) with a strong inhibitory effect against the plant pathogen *Alternaria brassicae* that can cause leaf spot of Chinses cabbage [11]. Pleosporalone A and B were isolated from Pleosporales sp. CF09-1, which was sampled from marine sediment in the Bohai Sea, and exhibited inhibitory activities against several plant pathogens, including *Rhizopus oryzae*, *Phytophthora capsica*, *Alternaria brassicicola*, *Botryosphaeria dothidea* and *Fusarium oxysporum* [12,13]. In addition, previous studies also documented that several marine-derived secondary metabolites from fungi displayed a high lethal effect on brine shrimp *Artemia salina* [14,15] and Lepidoptera pests such as *Helicoverpa armigera* [16,17] and *Spodoptera litura* [18,19]. These advances add to the importance of marine-derived fungi in the discovery of secondary metabolites applied to controlling agricultural pests and diseases. However, marine-derived fungi still represent an underestimated but rich source for bioactive screening and new secondary metabolites [3].

Supported by the investigation project of microbial resources in coastal tidal flats of China, we obtained many fungal isolates from marine macroalgae and intertidal sediment, and screened their insecticidal and antifungal activities using brine shrimp (*Artemia salina*), green peach aphid (*Myzus persicae*) and seven plant pathogens (e.g., *Alternaria alternata*, *Mucor hiemalis*) as targets. The aims of this study are (i) to identify the bioactivities of their secondary metabolites, and (ii) to screen the candidate fungal strains that have application potential in controlling agricultural pests and diseases.

## 2. Materials and Methods

### 2.1. Environmental Sampling and Fungal Isolation

Fungi associated with macroalgae and sediment collected from intertidal zones of Qingdao, China, were investigated using culture-dependent methods during 2020. Macroalgae were placed into sterile plastic bags and carried back to the laboratory. The uppermost 2–10 cm of marine sediment layers were sampled using sterile hollow glass tubes, which were sealed and stored in a freezer at 4 °C until processed within one month. Algae samples were washed with sterile seawater and immersed in 75% ethanol for one minute. Each algal thallus was cut into approximately 2 × 2 mm segments that were finely ground in sterile seawater with mortars and pestles. Finally, 100 μL of supernatant solution was spread over the culture media. For the isolation, 9 cm Petri dishes containing potato dextrose agar (PDA) supplemented with 1 g/L Penicillin G and Streptomycin sulfate were prepared. Petri dishes were then sealed, incubated at 20 °C and examined periodically. When colonies developed, they were transferred immediately to fresh media with PDA to obtain pure isolates. All pure isolates were stored at 4 °C in the dark.

### 2.2. Taxonomical Identification of Fungal Isolates

Morphological and molecular approaches were combined to taxonomically annotate fungal isolates, as previously mentioned by Cheng [20] and Wang [21]. Microscopic characteristics were observed from point-inoculated media in Petri dishes incubated for 14 days, and were measured from more than 50 individuals with an Olympus BX51 microscope (Tokyo, Japan).

Molecular identification of fungal isolates was conducted using a Megablast search of NCBI’s GenBank of nucleotide database with ITS rDNA that was amplified by the primers ITS1 and ITS4 [22] from extracted genomic DNA of fungal isolates. PCR reactions and sequencing were performed using the process described by Cheng [23]. All isolates and corresponding ITS rDNA sequences for molecular identification are shown in Table S1.

### 2.3. Fermentation

For chemical investigation, static cultivation of fungal strains was performed in liquid potato–dextrose broth medium (PDB, 1000 mL seawater, 20 g glucose, 5 g peptone, 3 g yeast extract, pH 6.5–7.0, liquid medium/flask = 300 mL) in 1 L Erlenmeyer flasks for 30 days at room temperature. An equal volume of EtOAc and CH_2_Cl_2_-MeOH was added to the flasks for extraction, then the crude extracts were obtained after vacuum concentration using the method suggested by Li [10].

### 2.4. Screening of Insecticidal Activities

**Figure 1:**
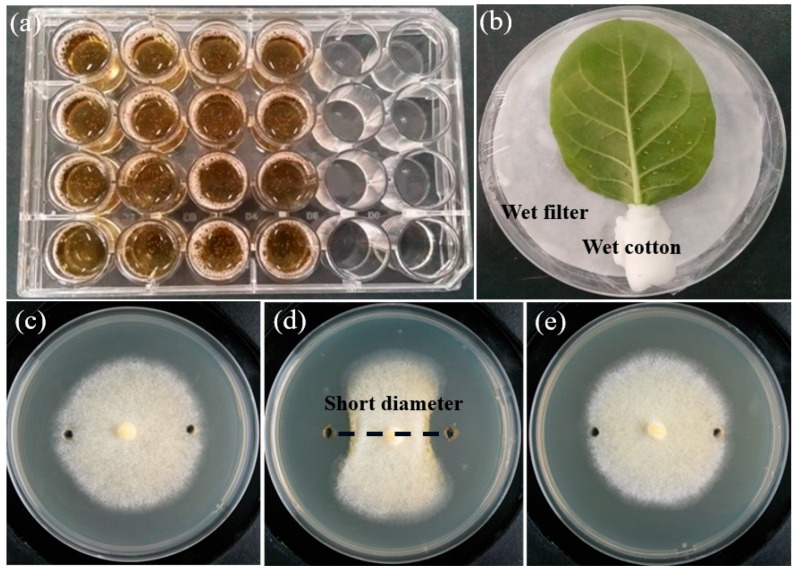
The methods adopted to test insecticidal and antifungal activities; 24-well cell culture plate for testing toxicity for brine shrimp (**a**); the aphids treated with an impregnation method in a Petri dish with moisturizing treatment (**b**); the modified micro-atmosphere method to check antifungal activity, treated with DMSO (**c**); extract crudes ((**d**), a case of AS67); and Prochloraz (**e**).

Insecticidal activities were conducted with two steps that included preliminary and secondary screenings using brine shrimp and green peach aphid, respectively. During preliminary screening, the supernatant of static cultivation of fungal strains after centrifugation was used to test brine shrimp toxicity according to Meyer [24] and Nazir [25] by using a tissue culture plate with 24-well cells. Briefly, about 1 mL of the fermentation supernatant was added to each cell filled with 1 mL of culture medium of brine shrimp (ca. 30 individuals) (Figure 1a), and then bred at 28 °C for checking mortality of brine shrimp after 4 and 24 h. PDB medium was used as negative control and each experiment was repeated four times. An adjusted mortality rate (AMR) was calculated with the following formula: AMR (%) = (mortality rate of the treated group ‒ mortality rate of the control group) × 100%/(survival rate of the control group).

The fungal isolates that exhibited a high AMR, more than 70% after 24 h on brine shrimp, were randomly selected for secondary screening on green peach aphid using a method of impregnation. The crude extracts were configured at the concentration of 10 mg/mL using acetone as the suspending agent to test aphid toxicity. The aphids (50 individuals with the same age and body size and reared in tobacco leaves) were immersed in the 10 mg/mL crude extracts for ten seconds. Then, these aphids were transferred into tobacco leaves that were placed in Petri dishes with moisturizing treatment (Figure 1b) for checking mortality after 24 and 48 h. Acetone was used as a negative control. Each experiment was repeated four times, and AMR was calculated as mentioned above.

### 2.5. Screening of Antiphytogenic Activity

Seven plant pathogens, which included *Alternaria alternata*, *Berkeleyomyces basicola* (syn. *Thielaviopsis basicola*), *Cochliobolus heterostrophus*, *Gaeumannomyces graminis*, *Glomerella cingulate*, *Mucor hiemalis* and *Phytophthora parasitica* var. *nicotianae* were selected as the targets to check antiphytogenic activity.

The crude extracts of marine-derived fungi were configured at two concentrations (10.0 and 1.0 mg/mL) using dimethyl sulfoxide (DMSO) as the suspending agent to test inhibitory effect on plant pathogens. The modified micro-atmosphere method [26] was adopted to assess antifungal activity of the crude extracts with modifications. For each plant pathogen, a piece of mycelial agar (diameter = 7 mm) from a 7-day culture was placed at the center of a Petri dish (diameter = 90 mm) containing 20 mL of PDA medium. Two holes (diameter = 6 mm) were symmetrically drilled into the agar plate with the distance of 15 mm from the edge of the dish. A fitted volume of the crude extracts dissolved in DMSO was added to the holes, and then the Petri dishes were incubated at 28 °C for five days to observe the inhibition zone. DMSO and a commercial fungicide, Prochloraz (a broad-spectrum fungicide, Sinopharm Chemical Reagent Co., Ltd., Shanghai, China), were used as negative and positive controls, respectively. Each experiment of bioactivity was repeated three times. The short colony diameters of plant pathogens treated by DMSO (SCDdmso), crude extract (SCDce) and Prochloraz (SCDp) (Figure 1c–e) were measured and used to calculate inhibitory rate (IR) using the following formula: IR (%) = (SCDdmso ‒ SCDce (or SCDp) × 100%/(SCDdmso). When necessary, unpaired two-sample t-test (one-way ANOVA) was used to explore variance between individual fungal strain and the positive control in terms of inhibitory rate.

## 3. Results

### 3.1. Fungal Diversity Involved in Bioactivity Screening

**Figure 2:**
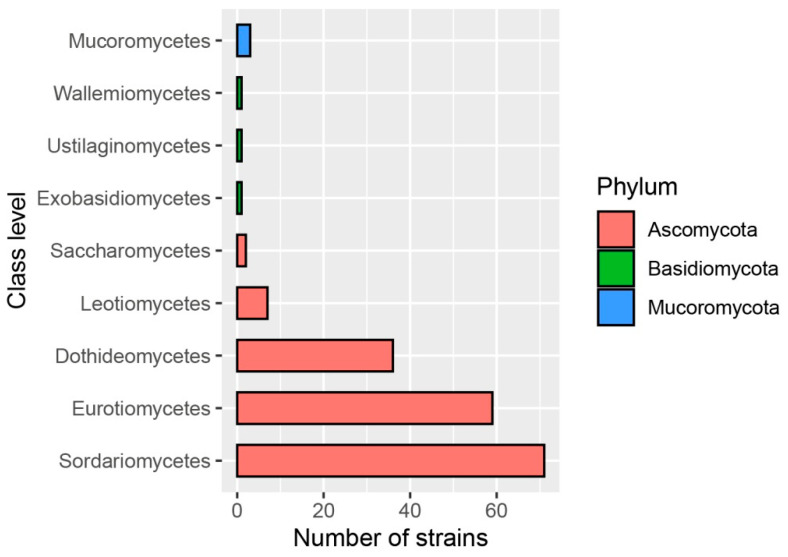
The number of fungal strains belonging to different classes that were isolated from intertidal zones of Qingdao.

In total, 181 fungal strains were isolated from marine algae (64 strains) and sediment (117 strains) from the Qingdao intertidal zones, and selected to test bioactivity. According to morphological and molecular features, a majority of them (175 strains, 96.7% of total strains) were identified as members of Ascomycota, spanning 5 known classes, 16 orders, 36 families and 59 genera (Table S1). Among them, Sordariomycetes (71 strains), Eurotiomycetes (59 strains) and Dothideomycetes (36 strains) appeared abundantly (Figure 2), mainly represented by common genera including *Acremonium* (8 strains), *Fusarium* (6 strains), *Trichoderma* (5 strains), *Aspergillus* (35 strains), *Cladosporium* (12 strains) and *Alternaria* (11 strains). In addition, six strains were affiliated with Basidiomycota (3 strains) and Mucoromycota (3 strains). At species level, 181 strains were represented by 134 fungal taxa, suggesting a high-level of fungal diversity in intertidal zones of Qingdao. Meta-screening of antimicrobial and insecticidal activities on these fungi could provide better overall insight into the bioactivity of marine-derived fungi.

### 3.2. Toxicity on Brine Shrimp and Green Peach Aphid

For brine shrimp, 85 fungal strains (47% of the total strains) displayed a high AMR of more than 70% at 24 h (Table 1, Figure S1). Among them, 14 strains exhibited an acute toxicity to brine shrimp with more than 75% of AMR at 4 h, especially seven strains with 100% of mortality, including AS13 (*Aspergillus niger*), AS65 (*Trichoderma scalesiae*), AS99 (*Fusarium verticillioides*), AS121 (*Emericella nidulans*), AS113 (*Geotrichum* sp.), AS133 (*Fusarium avenaceum*) and AS129 (*Penicillium simplicissimum*).

Different species within the same genus, even different strains within the same species, showed distinct toxicity to brine shrimp (Figure S1). For example, the AMR of AS73 (*Acremonium cellulolyticus*) was 100% at 24 h, whereas the AMRs of AS148 (*A. persicinum*) and AS85 (*A. potronii*) were zero. A similar situation occurred in two *Aspergillus* strains, AS11 (*A. gracilis*) and AS123 (*A. varians*). AS23 and AS109 were taxonomically identified as *Cladosporium colombiae*; however, the former exhibited an AMR of 94.9% at 24 h, and the latter did not show any toxicity.

**Figure 3:**
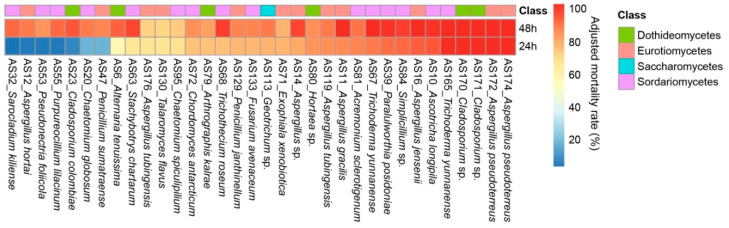
Toxicity of the crude extracts on peach aphid at a concentration of 10 mg/mL; only the 34 fungal strains with more than 70% of mortality after 48 h are shown here.

Forty-eight strains with more than 70% of AMR at 24 h on brine shrimp were selected to conduct the secondary round of screening on insecticidal activities using green peach aphid at a concentration of 10 mg/mL of crude extracts. Twenty-two strains exhibited an acute toxicity on aphid with more than 70% of AMR at 24 h, while seven stains, including AS12 (*Aspergillus hortai*), AS20 (*Chaetomium globosum*), AS23 (*Cladosporium colombiae*), AS32 (*Sarocladium kiliense*), AS47 (*Penicillium sumatraense*), AS53 (*Pseudonectria foliicola*) and AS55 (*Purpureocillium lilacinum*), exhibited nonacute toxicity with less than 20% of AMR at 24 h and more than 70% of AMR at 48 h, respectively (Figure 3). This difference in lethal duration might highlight distinct toxic modes among these strains on aphid.

### 3.3. Inhibitory Effect against Plant Pathogens

**Figure 4:**
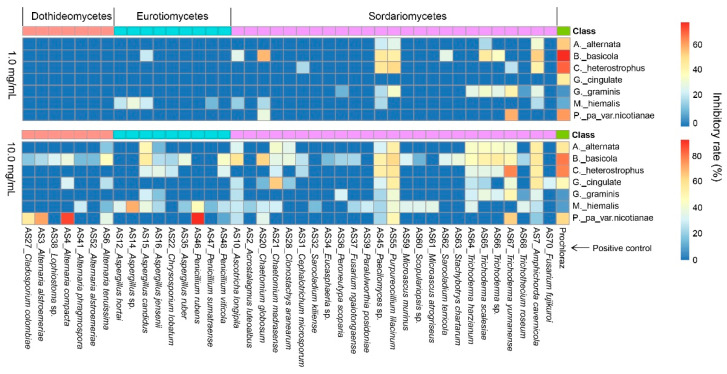
Inhibitory effects of 41 marine-derived fungal strains against seven plant pathogens with Prochloraz as positive control at 1.0 and 10.0 mg/mL concentrations, respectively. Four strains (AS33, AS40, AS50 and AS53) that had no observed effects are excluded.

Among the 45 fungal strains screened for antifungal activities, most exhibited a certain degree of inhibitory effect on seven plant pathogens at the two concentrations, 10.0 and 1.0 mg/mL (Figure 4). At 10.0 mg/mL concentration, the two strains of AS7 (*Amphichorda cavernicola*, IR = 54.11%) and AS55 (*Purpureocillium lilacinum*, 52.66%) were equal to the positive control Prochloraz (56.52%) (*p* > 0.05) in the terms of inhibitory effect on the plant pathogen *Alternaria alternata*. For the plant pathogen *Berkeleyomyces basicola*, stronger inhibitory effects were found in two *Alternaria* strains AS3 (*A. alstroemeriae*, 72.27%) and AS4 (*A. compacta*, 87.75%) and the *Penicillium* strain AS46 (*P. rubens*, 92.26%) compared to Prochloraz (61.95%) (*p* < 0.05). For *Cochliobolus heterostrophus*, AS20 (*Chaetomium globosum*, 63.00%) and AS55 (*P. lilacinum*, 62.44%) showed relatively higher inhibitory effects than other fungal strains, but less than Prochloraz (79.56%) (*p* < 0.05). For *Glomerella cingulate*, the *Trichoderma* strain AS67 (*T. yunnanense*, 77.72%) had an equal effect with Prochloraz (77.72%). For *Gaeumannomyces graminis*, three strains, including AS7 (*A. cavernicola*, 55.58%), AS21 (*Chaetomium madrasense*, 65.36%) and AS55 (53.61%), exhibited stronger effects than Prochloraz (48.37%).

Interestingly, Prochloraz showed poor inhibitory effect against the two pathogens *Mucor hiemalis* and *Phytophthora parasitica* var. *nicotianae* that could be strongly inhibited by AS67 (*Trichoderma yunnanense*, 49.40%) and AS14 (*Aspergillus* sp., 70.37%), respectively. The two marine-derived fungi also showed inhibitory effects to a certain extent on these pathogens at the concentration of 1.0 mg/mL (Figure 4). Furthermore, at the concentration of 1.0 mg/mL, there were four strains including AS7 (*Amphichorda cavernicola*), AS20 (*Chaetomium globosum*), AS55 (*Purpureocillium lilacinum*) and AS67 (*T. yunnanense*) with more than 50% of IR on one of the seven pathogens.

Combining the results from both insecticidal and antifungal experiments, five fungal strains, including AS7 (*Amphichorda cavernicola*), AS14 (*Aspergillus* sp.), AS21 (*Chaetomium madrasense*), AS55 (*Purpureocillium lilacinum*) and AS67 (*Trichoderma yunnanense*), exhibited an equal or higher effect against one or two plant pathogens, and more than 75% or even 100% lethality on brine shrimp at 24 h.

## 4. Discussion

Diverse fungal taxa have been reported from various habitats including coastal waters, the open ocean, and the deep biosphere [5,27,28,29]. It is estimated that there are 10,000 or more species of fungi that inhabit the ocean [30]. A recent review listed 1257 marine fungal taxa recorded from a wide diversity of habitats (e.g., mangrove, salt marsh, deep-sea) and substrates (e.g., drifting wood, sediment, plants, animals, algae) and occurring as saprobes, symbionts, parasites or mutualists [31]. The intertidal zone is well represented by a broad range of habitats such as mangrove, sand beach, estuarine, shallow coral and salt marsh that host rich fungal biodiversity and high endemicity [5]. For example, researchers have isolated about 500 fungal taxa from salt marsh worldwide and members of Ascomycota are dominant, mainly represented by Dothideomycetes and Sordariomycetes [32]. Similarly, almost all isolates (175 isolates, accounting for 96.7% of total isolates) were taxonomically annotated as the taxa of Ascomycota in this study. A large fraction of these species belongs to *Acremonium*, *Aspergillus*, *Cladosporium* and *Penicillium*, which are ubiquitous in both the oceanic and terrestrial environments [27,31]. Moreover, some isolates such as AS18 (*Neodevriesia* sp., 92.20% sequence similarity with *N. metrosideri* (NR_161141.1)), AS86 (*Peroneutypa* sp., 95.17% with *P. rubiformis* (NR_158867.1)) and AS126 (*Gamszarea* sp., 93.19% with *G. humicola* (NR_172830.1)) may present new species due to the low sequence similarity with reference taxa in the NCBI nucleotide database. Our results support the idea that intertidal areas host rich fungal diversity that needs further investigation.

Brine shrimp bioassay is considered as a rapid preliminary screening for an effective presence of biochemical activity and was used to determine the toxicity of crude extract or its chemical compound [25,33]. Previous studies have revealed that chemical compounds extracted from marine-derived fungi such as the members of *Aspergillus*, *Eurotium*, *Penicillium*, *Stemphylium* exhibited a significant toxicity on brine shrimp [14,15,34,35]. In this study, the supernatants of static cultivation of 85 fungal strains (accounting for 47.0% of all treated fungal strains) isolated from marine algae and sediments in the Qingdao intertidal zones were found to be toxic to brine shrimp, as characterized by more than 70% of lethality after 24 h. Additionally, 14 strains (more than 75% mortality at 4 h) and 49 strains (<10% of mortality at 4 h, >70% at 24 h) were acute-toxic and nonacute-toxic to brine shrimp, respectively, indicating diverse modes of action for secondary metabolites produced by marine-derived fungi. During the secondary screening, there were 34 fungal strains that exhibited high toxicity (>70% mortality at 48 h) of their crude extracts on peach aphid, an important agricultural pest. Our results further highlight the broad potential for marine-derived fungi as candidate resources to explore bioactive compounds [36].

Marine algicolous fungi have attracted great attention for natural product research during the past two decades due to their chemical diversity and biological activity [4,36]. Large amounts of secondary metabolites, which feature cytotoxic, antioxidative, brine shrimp-toxic, antimicroalgal, enzyme-inhibitory, antibacterial and antifungal activities, have been isolated and identified from numerous fungal genera including *Aspergillus*, *Penicillium*, *Chaetomium*, *Fusarium*, *Phoma*, *Trichoderma*, *Acremonium*, *Alternaria*, *Beauveria* and *Cladosporium* [4,6,10]. However, few chemical investigations associated with antifungal and insecticidal activities were conducted on some genera, such as *Acremonium*, *Alternaria*, *Chaetomium*, *Fusarium* and *Phoma*. Our results found that members of these fungal genera sampled from marine algae of Qingdao intertidal zones displayed antifungal bioactivities and strong toxicity toward brine shrimp. For example, two strains (AS3, AS4) of *Alternaria* and one strain (AS21) of *Chaetomium* showed higher inhibitory effects on the plant pathogens *Berkeleyomyces basicola* and *Gaeumannomyces graminis*, respectively, compared to the fungicide Prochloraz. Similar strong inhibitory effects can be found in the two newly recorded marine algicolous species, *Amphichorda cavernicola* (AS7) and *Purpureocillium lilacinum* (AS55), against the plant pathogen *G. graminis*. These findings further support the idea that marine algicolous fungi are valuable resources for exploring agricultural chemicals with antifungal and insecticidal activities [4,8], especially with a perspective toward searching for new metabolic products.

## 5. Conclusions

In this study, 181 fungal strains were isolated from the intertidal zones of Qingdao, and screened for insecticidal and antifungal activities. About half of the total strains exhibited either a high toxicity or inhibitory effect on brine shrimp, green peach aphid and plant pathogens, and five strains affiliated with *Amphichorda* (AS7), *Aspergillus* (AS14), *Chaetomium* (AS21), *Purpureocillium* (AS55) and *Trichoderma* (AS67) showed simultaneously efficient insecticidal and antifungal activities. Our findings indicate diverse bioactivities of marine-derived fungi and a great exploration potential for their application in controlling agricultural pests and pathogens.

## Figures and Tables

**Table 1:** Toxicity on brine shrimp of the static cultivation supernatants of 181 marine-derived fungal strains at 24 and 4 h. The 85 strains showed here at 4 h were those with more than 70% of adjusted mortality rate (AMR) at 24 h.

Test Time	AMR (%)	Number of Strains	Tested Strains	Proportion (%)
24 h	>70	85	181	46.96
4 h	=100	7	85	8.24
	>75	14	85	16.47
	<10	49	85	57.65
	=0	25	85	29.41

## Data Availability

Not applicable.

## Supplementary Materials

The following supporting information can be downloaded at: https://www.mdpi.com/article/10.3390/jof8121240/s1, Figure S1: Acute and nonacute toxicities of the supernatants of the 85 fungal strains on brine shrimp after 24 h with more than 70% of an adjusted mortality rate; Table S1: Information for the 181 fungal strains.
