# Supplementary figures and images for: Screening of Insecticidal and Antifungal Activities of the Culturable Fungi Isolated from the Intertidal Zones of Qingdao, China
Source: J Fungi (Basel). 2022 Nov 24;8(12):1240. doi: 10.3390/jof8121240 (PMC9783798; doi:10.3390/jof8121240)

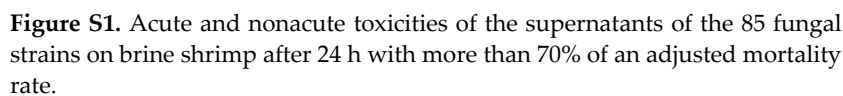

Supplement: Supplementary file 1 [file jof-08-01240-s001.zip › jof-2037108-supplementary-Figure S1.pdf]
